# Tubulin βII and βIII Isoforms as the Regulators of VDAC Channel Permeability in Health and Disease

**DOI:** 10.3390/cells8030239

**Published:** 2019-03-13

**Authors:** Marju Puurand, Kersti Tepp, Natalja Timohhina, Jekaterina Aid, Igor Shevchuk, Vladimir Chekulayev, Tuuli Kaambre

**Affiliations:** Laboratory of Chemical Biology, National Institute of Chemical Physics and Biophysics, Akadeemia tee 23, 12618 Tallinn, Estonia; kersti.tepp@kbfi.ee (K.T.); natalja.timohhina@kbfi.ee (N.T.); jekaterina.aid@gmail.com (J.A.); igor@chemnet.ee (I.S.); vladimir@chemnet.ee (V.C.); tuuli.kaambre@kbfi.ee (T.K.)

**Keywords:** tubulin, voltage-dependent anion channel (VDAC), mitochondria, hexokinase, oxidative phosphorylation, creatine kinase, oxidative muscle, brain, synaptosomes, cancer

## Abstract

In recent decades, there have been several models describing the relationships between the cytoskeleton and the bioenergetic function of the cell. The main player in these models is the voltage-dependent anion channel (VDAC), located in the mitochondrial outer membrane. Most metabolites including respiratory substrates, ADP, and Pi enter mitochondria only through VDAC. At the same time, high-energy phosphates are channeled out and directed to cellular energy transfer networks. Regulation of these energy fluxes is controlled by β-tubulin, bound to VDAC. It is also thought that β-tubulin‒VDAC interaction modulates cellular energy metabolism in cancer, e.g., switching from oxidative phosphorylation to glycolysis. In this review we focus on the described roles of unpolymerized αβ-tubulin heterodimers in regulating VDAC permeability for adenine nucleotides and cellular bioenergetics. We introduce the Mitochondrial Interactosome model and the function of the βII-tubulin subunit in this model in muscle cells and brain synaptosomes, and also consider the role of βIII-tubulin in cancer cells.

## 1. Introduction

The beta-tubulins, as subunits of tubulin (αβ-tubulin heterodimers), the building block of microtubules (MTs), are a widespread cytoskeletal proteins present in all eukaryotic cells. Nowadays, in humans 10 α-tubulin and nine β-tubulin genes have been identified in the tubulin gene family [1]. At the level of gene expression, it has been found that genes TUBB2A (IIα), TUBB2B (IIβ), TUBB3 (IIIβ), and TUBB4 (IVα) have high expression in the brain; TUBB2C (IVβ) in the heart and skeletal muscles. However, the contribution of the different isotypes to the total beta-tubulin content varied for each tissue and had a complex pattern [1]. Tumor cells are characterized by a great increase in expression of TUBB3, while TUBB6 (V) expression was largely decreased in most tumors [2].

Both α- and β subunits have a globular shape with an unstructured C-terminal tail. They vary mostly in the length and primary sequence of the disordered anionic C-terminal tails (CTTs), which is also the most variable part in the tubulin protein sequence [3]. It consists of 10–15 amino acids, many of which are strongly anionic, and projects out from or collapses onto the surface of MTs [4]. While the globular parts of tubulin can polymerize into microtubules, CTTs are free to interact with membranes or proteins. Tubulins are also the target of protein post-translational modifications. Modifications on the CTTs include mono- and polyglutamylation, mono- and polyglycylation, and tyrosination/deyrosination. The ‘tubulin code’ hypothesis assumes that specific tubulin isotypes or post-translational modifications confer unique biochemical activities [5]. However, the roles of these different tubulin isotypes remain poorly understood.

Tubulins are involved in several functions in a cell, including structural responsibilities in the polymerized form (microtubules) as well as regulatory roles in unpolymerized tubulin form [3]. Microtubules are responsible for the mitochondrial subcellular arrangement and dynamics in skeletal and heart muscle cells. Their cytoarchitecture, isoform composition, interaction partners, and post-translational modifications act as spatial cues to navigate mitochondria within the cellular space and help to stall and anchor mitochondria at sites of high energy demand [6]. However, the bioenergetics regulatory role of unpolymerized tubulin may be related to tuning the permeability of the voltage-dependent anion channel (VDAC) located in the mitochondrial outer membrane (MOM). 

Mitochondrial VDAC plays a key role in maintaining high rates of oxidative phosphorylation (OXPHOS) as well as in the realization of apoptotic programs [7,8,9]. Moreover, the formation of the mitochondrial membrane potential depends on the flux of respiratory substrates, ATP, ADP, and Pi through VDAC. Thereby, the mitochondrial function is strongly related to VDAC permeability to adenine nucleotides. In oxidative skeletal muscles and brain synaptosomes tubulin βII isoform is described as serving as a regulator of mitochondrial function via tuning VDAC permeability to adenine nucleotides. The ability to block VDAC is also considered to be an activity of βIII isoform in cancer cells. The detailed evaluation of VDAC‒tubulin CTT in artificial membranes by using yeast αβ-tubulin constructs with distinct human β- and α-tubulin CTTs confirmed that CTTs of human tubulin βII and βIII isoform and α-tubulin I are able to block VDAC [3]. Replacement of the terminal alanine with a tyrosine on the β-tubulin CTT peptide grafted to albumin reduced construct‒VDAC binding activity. Removal of C-terminal tyrosine from the α-tubulin CTT peptide‒albumin construct significantly enhanced VDAC closure activity of the peptide, and activation is reversed by subsequent removal of the of the glutamic acid penultimate to the tyrosine [10]. These results indicate that the interaction between the tubulin dimer and VDAC is determined by the CTTs and that a small change in sequence or post-translational modification, especially tyrosination/detyrosination, of these tails results in substantial changes to the VDAC closure. How other possible post-translational modifications in tubulin CTT affect the tubulin‒VDAC interaction requires further investigation.

In cells with a high and frequently changing energy demand, like muscle cells, the OXPHOS system is organized into large protein complexes; one of them is the Mitochondrial Interactosome (MI) [11,12]. MI is a large transmembrane complex consisting of an ATP synthasome, mitochondrial creatine kinase (MtCK) or other representatives of mitochondrial kinases, VDAC, and some protein factors, which regulate the MOM permeability for adenine nucleotides. Profound changes occur in the MI composition of cardiomyocytes during postnatal development and less with aging [13,14]. The changes in the composition and function of MI in malignancy have been insufficiently investigated. There are several significant consequences for restrictions of adenine nucleotide intracellular diffusion, including low VDAC permeability to ATP and ADP. To overcome the difficulties in energy transport, cardiomyocytes and other highly oxidative muscles have developed mechanisms such as metabolite micro-compartmentalization, channeling of metabolites, and functional coupling of enzymes into supramolecular complexes. Most common phosphotransfer networks consisted of creatine and adenylate kinase isoforms to ensure transport of high-energy phosphate without any need for ADP and ATP to diffuse all over the cell. In addition to the use of energy transport networks for channeling ATP to ATP-ases, these networks also provide a precise feedback system between the sites of energy consumption and OXPHOS [15,16,17,18].

Studies of VDAC reconstituted into planar lipid bilayers, as well as model experiments with isolated mitochondria, show that tubulin strongly decreases the conductance of VDAC towards some adenine nucleotides (ADP/ATP). Therefore, it has been proposed that this phenomenon could play a central role in the regulation of mitochondrial function in both normal and tumor cells [19,20].

In this article we review the function of unpolymerized βII- and βIII-tubulin as part of the MI complex in muscle cells and brain synaptosomes, and in cancer cells. 

## 2. Tubulin as the Regulator of VDAC Permeability in Oxidative Skeletal and Heart Muscles

Studies published in 1995‒1996 show that different muscle types have different diffusion restrictions for adenine nucleotides through the MOM and that specific mitochondria-cytoskeleton interactions could be responsible for this feature [21,22,23].

These papers describe the kinetics of in vivo regulation of mitochondrial respiration by ADP in saponin-skinned fibers of rat cardiac muscle, slow twitch skeletal muscles (oxidative muscles with red fibers) skeletal *m. soleus* and fast-twitch skeletal muscles (glycolytic muscles with white fibers) like *m. gastrocnemius*, *m. plantaris*, *m. quadriceps* and *m. tibialis anterior*. The main result of these studies is that the kinetics of respiration regulation in muscle cells is tissue-specific. Treatment of muscle fibers with proteolytic enzymes significantly decreased the diffusion restrictions for ADP, measured as an apparent Michaelis-Menten constant for exogenous ADP (KmADP), in cardiac and *m. soleus* skinned fibers without significant alteration in maximal respiration (Vmax) or in MOM intactness. Rat slow-twitch muscles have a high apparent KmADP value compared to studied fast-twitch muscles (300‒400 µM and 10‒20 µM, respectively) (Table 1). Moreover, the reduction of the apparent KmADP to 40‒100 µM by trypsin treatment indicated that respiration in slow-twitch muscles is controlled by a cytoplasmic protein not expressed in fast-twitch muscles. These results were confirmed in many subsequent papers [12,20,23,24,25,26,27,28,29,30]. In addition, it has been shown that isolated mitochondria from slow-twitch and fast-twitch muscles display similar characteristics. All these results show that regular intracellular arrangement of mitochondria and high apparent KmADP are most probably related phenomena, due to the presence of proteins sensitive to trypsin. These proteins seem to be connected to the cytoskeleton, since similar high apparent KmADP values are also observed for “phantom” cells and fibers, from which myosin has been extracted and that contain mostly mitochondria, sarcoplasmic reticulum and cytoskeletal structures [31,32]. Further, the microtubular network, desmin and plectin as three important cytoskeletal components were taken into consideration as possible regulators of MOM permeability. The attempts to provide major regulatory role to desmin by using desmin knock-out mice has not yielded clear results [32]. More detailed investigation of the proteolytic treatment revealed that destruction of microtubular and plectin networks affect the control of mitochondrial function in vivo [31]. 

The first attempts to identify this presumably cytoskeletal protein as tubulin have failed, since colchicine and taxol were not found to change high values of the apparent KmADP in skinned slow-twitch muscle fibers [24]. Possible breakthrough in solving the long-standing puzzle came with the work by Rostovtseva et al. 2008 in which they showed that nanomolar concentrations of α and β tubulin heterodimers induce voltage-sensitive reversible closure of VDAC, reconstituted into planar phospholipid membranes. Moreover, tubulin added to isolated brain and cardiac mitochondria decreased ADP availability to mitochondria (increase the KmADP) and partially restored the high apparent KmADP found in permeabilized cardiac cells [12,20]. Indeed, studies of gene expression and cytoskeleton organization in mice and rat heart and skeletal muscles demonstrated that tubulin network could be responsible for respiration regulation and mitochondrial organization in oxidative striated muscle cells. Tubulin was a good candidate for functioning as a modulator of mitochondrial permeability in oxidative muscle cells because interactions of mitochondria with tubulin have been observed by many authors [33,34]. First, it was found that β-tubulin cDNA (β-tubulin gene M-beta-4) is present in mouse myocardium and oxidative *m. soleus* but absent in glycolytic *m. extensor digitorum longus* [35,36]. Further analysis of cardiac cells has revealed regular arrangement of βII tubulin (fully co-localized with mitochondria) [37,38], βIV-tubulin demonstrated a characteristic staining of branched network, βIII-tubulin was matched with Z-lines, and βI-tubulin was diffusely spotted and fragmentary polymerized. Microtubular network formed by βIV-tubulin isotype in cardiac muscle cells is the probable cytoskeletal backbone interconnecting mitochondria via βII-tubulin, sarcomere via βIII-tubulin, membranes (sarcolemma, sarcoplasmic reticulum) via βI-tubulin and other structures [39]. The highly specific distribution of different isotypes of β-tubulin in adult cardiomyocytes allows us to suppose that they are complementarily related with each other and with intracellular organelles. The importance of precise intracellular structural arrangement in regulation of energy metabolism is evidenced by fact that during postnatal development of rat cardiomyocytes the apparent KmADP value correlates with Pearson coefficient for colocalization of βII-tubulin and VDAC. Thus, the functional maturity on the level of mitochondrial respiration regulation is achieved after the maturation of intracellular cytoarchitecture [13]. Furthermore, rat cardiomyocytes preserve this co-distribution of βII-tubulin and VDAC during aging [14]. Similar to the cardiac muscle in oxidative skeletal muscle *m. soleus* the high apparent KmADP value is associated with high expression of non-polymerized βII-tubulin. In addition, very low expression of non-polymerized form of βII-tubulin in glycolytic muscles like *m. extensor digitorum longus* and *m. gastrocnemius* white is associated with high MOM permeability for adenine nucleotides (low apparent KmADP) [30]. These results support a model whereby energy metabolism is directly linked to the highly organized intracellular architecture in cardiomyocytes and slow-twitch skeletal muscles; this βII-tubulin isoform may participate in the regulation of VDAC permeability in oxidative muscle cells. However, it cannot be discounted that other β-tubulin or α-tubulin isoforms could also bind to VDAC and influence its conductance. At present, the distribution of α-tubulins in muscle cells is totally unknown, and it is also unclear whether post-translational tubulin modifications could influence the interaction of tubulin with VDAC.

**Figure 1:**
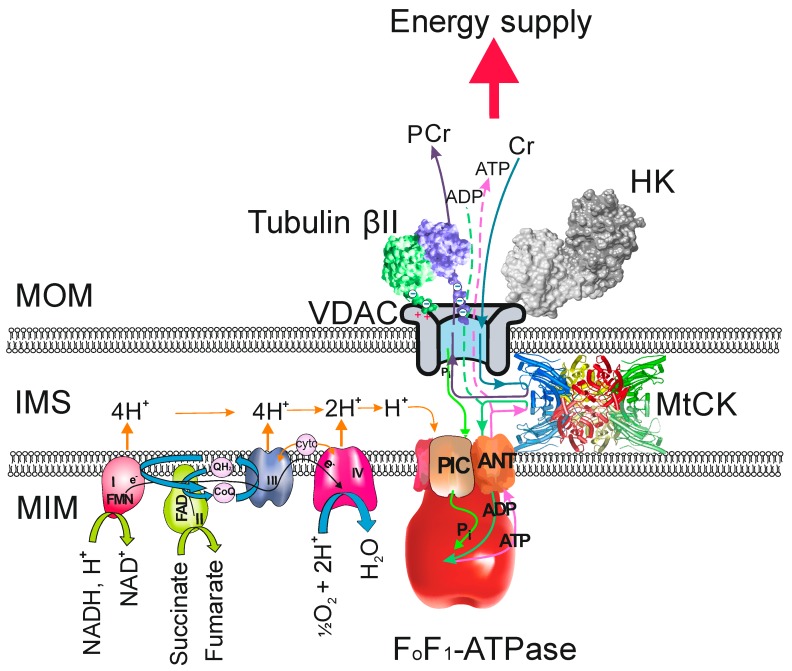
The Mitochondrial Interactosome (MI) and the role of tubulin in its regulation. In the MI of oxidative muscle cells the tubulin C-terminal tail interacts with the mitochondrial voltage-dependent anion channel (VDAC) and restricts the diffusion of adenine nucleotides ATP and ADP through the channel. Tubulin-VDAC interaction does not interfere with creatine (Cr) and phosphocreatine (PCr) movement in or out of the mitochondria. Creatine is phosphorylated in the mitochondrial intermembrane space (IMS) by mitochondrial creatine kinase (MtCK). As the MtCK is located close to the adenine nucleotide translocase (ANT), the ATP produced in the mitochondrial matrix is quickly directed to MtCK reaction and ADP is transported back to the matrix. Due to this VDAC-tubulin interaction the energy-rich phosphate is directed to the CK energy transfer network and MtCK has become an important regulator of the ATP synthasome (consisting of the F_o_F_1_-ATPase, respiratory chain complexes, inorganic phosphate carrier (PIC) and ANT). Hexokinase (HK) can also bind to VDAC. The main task of this interaction is to guide the ATP produced in mitochondria to be available for glycolysis. In MI of cancer cells MtCK probably does not participate in the regulation of oxidative phosphorylation. To what extent and how tubulin (supposedly βIII-tubulin) and HK compete for binding on VDAC needs further investigation. MOM—mitochondrial outer membrane; MIM—mitochondrial inner membrane.

Remarkably, co-localization of βII-tubulin and VDAC and high apparent KmADP value in muscles is functionally related to ability of creatine to stimulate OXPHOS due to functional coupling between mitochondrial creatine kinase (MtCK) and adenine nucleotide translocase (ANT) [30]. MtCK is located at the outer surface of mitochondrial inner membrane in close vicinity of ANT [18,40] and the ADP formed in MtCK reaction is released into mitochondrial intermembrane space and may either return to matrix via ANT or leave mitochondria through VDAC (Figure 1) [41]. The flux distribution between these two routes depends on the permeability of this channel for adenine nucleotides. In permeabilized cardiomyocytes and slow-twitch skeletal muscle fibers addition of creatine increases mitochondrial apparent affinity to ADP and the apparent KmADP decreases to 80–100 µM in the presence of creatine [21,42,43]. In these muscle cells, through its binding with VDAC, βII-tubulin may support restriction of adenine nucleotides diffusion but there are no restrictions for phosphocreatine and creatine [12]. In the presence of creatine even low concentrations of added ADP, compared to a situation without creatine, can stimulate respiration. This is due to the working MI supercomplex where the signal of ADP is amplified by MtCK reaction. As ADP and ATP cannot leave from intermembrane space due to the diffusion restrictions caused by βII-tubulin–VDAC interaction, ATP is used for phosphocreatine production in the intermembrane space and the ADP is quickly channeled back to mitochondrial matrix as substrate for OXPHOS (Figure 1). Such of channeling high-energy phosphate to the creatine kinase network in the heart muscle provides also precise feedback signaling between energy consumption and production in conditions where energy consumption may suddenly increase several times [15,16,40]. Thereby, alterations in MI components and function could be as one of the possible mechanisms underlying cardiac diseases like ischemia-reperfusion injury. In the rat heart ischemia reperfusion injury induced heterogeneous intracellular rearrangement of βII-tubulin accompanied with decrease in KmADP [44]. Altogether, there is clear evidence that non-polymerized βII-tubulin is the factor regulating VDAC permeability for adenine nucleotides in oxidative muscle cells. As a consequence of the βII-tubulin–VDAC interaction the regulation of mitochondrial respiration by creatine in adult cardiomyocytes is accomplished via cooperatively functioning proteins such as ATP synthasome consisting of ATP synthase, respiratory system and inorganic phosphate transporter, MtCK, VDAC and tubulin that form a supercomplex called Mitochondrial Interactosome (Figure 1) [11,12,45]. How different diseases and conditions affect the composition and function of MI in the heart and skeletal muscles has not been sufficiently studied.

## 3. Tubulin as the Regulator of VDAC Permeability in Brain Synaptosomes

The complexity of neuronal cytoarchitecture requires highly organized regulation of the energy transport. During brain development microtubule cytoskeleton must mediate neurogenesis, neuronal migration and neuronal differentiation. Studies of synaptosomes nearly 15 years ago showed that in the presynaptic terminus alpha and beta tubulin subunits are specifically restricted to the equatorial microtubular coil [55,56]. Synaptic vesicles and synaptosomal plasma membranes mainly contained α-tubulin, whereas the β subunit was less abundant [57]. 

The neuronal microtubule array is highly complex, as reflected in the genetic and chemical diversity of the αβ-tubulin dimer through the expression of multiple α-tubulin and β-tubulin isoforms as well as chemically diverse and abundant post-translational modifications that are temporally and spatially regulated [5]. It is proposed that the expression pattern of a tubulin isoform might reflect the role of the microtubule cytoskeleton in that particular cell type at a given point in time [58,59]. The precise role of tubulins and the complete isoform printout of tubulins in regulating brain energy metabolism have not been sufficiently studied. 

In the brain, like in other excitable tissues with high and intermittent energy demands, CK network is present. In the brain it consists of the brain-type cytosolic CK (BB-CK), which is co-expressed with the ubiquitous mitochondrial isoenzyme, uMtCK [60,61]. Like in oxidative muscles, apparent KmADP measured in permeabilized synaptosomes in situ was rather high (110 ± 11µM) in comparison to that in isolated brain mitochondria (9 ± 1 µM). Also, the apparent KmADP decreased to 25 ± 1 µM in the presence of 20 mM creatine. This indicates that in brain synaptosomes, like in muscles, mitochondria have diffusion barriers for adenine nucleotides as well as functional coupling between MtCK and OXPHOS [47]. Moreover, in in vitro studies where isolated mitochondria were incubated with αβ-tubulin fraction from the rat or bovine brain, the combination drastically increased the apparent KmADP from 9 ± 1 µM to 169 ± 52 µM showing that in the rat brain, particularly in synaptosomes, MOM permeability for ADP, and ATP may be restricted by tubulin binding to VDAC [47]. Class II β tubulin, is the major brain β-tubulin isotype in adult brain cells, represented by high expression of TUBB2A (βIIa), TUBB2B (βIIb). Also, TUBB3 (βIII), and TUBB4 (βIVa) are highly expressed [2]. However, which tubulin isoforms participate in the regulation of MOM permeability to ATP and ADP in brain synaptosomes is not known.

## 4. The Regulatory Aspects of Tubulin on VDAC Permeability in Cancer Cells

During malignant transformation, cells acquire abnormal metabolism, whereby glucose is metabolized by aerobic glycolysis, as first described by Otto Warburg and now called the Warburg phenomenon, i.e., elevated glycolysis even in the presence of oxygen [62,63,64]. Now it is recognized that mitochondrial energy metabolism is as important as glycolysis to cancer development and survival [65]. The contribution and regulation of OXPHOS in cancer cells has also become an important research topic in recent years. Although altered expression of tubulin isotypes has been observed in a range of cancers (reviewed in [66]) the role of tubulin in regulation of mitochondrial outer membrane permeability of malignant cells is not clear. Several studies have shown that during formation of malignancy VDAC permeability for ADP is altered [48,49,52,67,68,69]. Recently, VDAC closure was hypothesized to contribute to the suppression of mitochondrial metabolism in the Warburg phenomenon [64]. There are two possible mechanisms proposed for how the MOM permeability for adenine nucleotides is regulated in cancer cells (Figure 1). According to the Warburg‒Pedersen model, highly expressed hexokinase (HK) binds to VDAC to suppress mitochondrial function while stimulating glycolysis. If HK-2 is bound to VDAC, then ATP from mitochondria will be guided directly to active sites on HK-2. As a consequence, the aerobic glycolysis is facilitated and malignant metabolic reprogramming occurs [68,70]. The second mechanism described in publications by Maldonado and co-workers propose that inhibition of VDAC by free tubulin limits mitochondrial metabolism in cancer cells. They found that in hepatocarcinoma cells an increase of free tubulin leads to depolarization of mitochondria, whereas a decrease, on the contrary, caused hyperpolarization. Additionally, free tubulin, protein kinase A and glycogen synthase kinase 3β dynamically regulate mitochondrial function in cancer cells but not in untransformed primary cells [71]. It can be hypothesized that blockage of the VDAC–tubulin switch may increase mitochondrial metabolism in cancer cells and leads to decreased glycolysis and oxidative stress that promotes mitochondrial dysfunction, bioenergetic failure, and cell death [72].

It is expected that βII-tubulin is involved in the genesis and development of many types of tumors like in early lymph node micrometastasis of colorectal cancer, neuroepithelial and brain tumors [52,73]. Thus, it can be hypothesized that free βII-tubulin competes with HK-1 or HK-2 for the binding sites on VDAC in order to regulate the aerobic glycolysis in tumor cells. To test this hypothesis Klepinin et al. examined the expression and possible co-localization of mitochondrial VDAC with HK-2 and βII-tubulin, the role of depolymerized βII-tubulin and the effect of both proteins in the regulation of MOM permeability in mouse sarcoma (HL-1) cells, murine neuroblastoma (N2a) cells and retinoic acid—differentiated N2a cells (dN2a). They demonstrated that βII-tubulin plays a minor role in regulation of energy metabolism in HL-1 murine sarcoma cells, in contrast to cardiac and slow-twitch skeletal muscles [52]. In HL-1 cells most of βII-tubulin was present in the nonpolymerized form and only some portion of this protein was associated with MOM [52]. In undifferentiated N2a (uN2a) cells significant changes in β-tubulin expression and intracellular distribution occurred during differentiation. In dN2a cells the intracellular content of βI- and βIII-tubulin was decreased, while βII-tubulin remained at the same level as in uN2a cells. In uN2a cells, βI-, βII- and βIII-tubulins were localized predominantly around the cell nucleus, whereas in dN2a cells a part of β-tubulin isotypes were assembled in filamentous structures that crossed the entire cell and neurites. The confocal microscopy of immunostained preparations of uN2a and dN2a cells revealed a similar degree for the HK-2‒VDAC colocalization. Also, no difference found in oxygraphic analysis of functional coupling between HK-2 and OXPHOS, indicated that differentiation of N2a cells had no effect on the binding of HK-2 to VDAC [52]. It can be concluded that in cancer cells βII-tubulin does not compete with HK for the binding sites on VDAC. One possibility is that another tubulin isoform other than βII participate in the regulation of MOM. In dN2a cells in comparison with undifferentiated cells both βI- and βIII-tubulin expression is significantly lower. Therefore, the candidate for the VDAC regulatory protein in neuroblastoma cells and also in other cancer cells could be βIII-tubulin (TUBB3).

The tubulin βIII subunit is prominently expressed during fetal and postnatal development of the brain [60,74]. Also, in neuroblastoma cell line SK-N-SH the mitochondrial membrane is found to be enriched in the tubulin-βIII isotype as compared with the cytosolic tubulin fraction [34]. However, tubulin-βIII overexpression has been reported in several tumors like rectal carcinoids and carcinoids of the small intestine [75], gastric cancer [76], colorectal cancer [50], gliomas [74], lung, renal, ovarian cancers [77], and lymphomas [78]. Moreover, the tumor drug resistance seems to be related to tubulin isoform expression profiles [66], especially βIII-tubulin overexpression [79]. Interestingly, this tubulin isoform was identified as a potential biomarker for high-grade aggressive tumors [80]. Determination of the expression of microtubule components may have prognostic value in solid tumors [81].

It is suggested that βIII-tubulin regulates cellular metabolism and glucose stress response signaling to promote cell survival and decrease the reliance of cells on glycolytic metabolism [82]. In cancer cells, βIII-tubulin with specific post-translational modifications was reported to localize in the mitochondrial membranes [82]. How and to what extent mitochondrial and cytosolic βIII-tubulin variants are related to the formation and progression of malignancies and to drug resistance needs further investigation.

Mitochondria from human breast and colorectal cancer exhibit an increased affinity for exogenous ADP compared to normal oxidative type tissues, but this value is still 6‒8 times higher than the apparent KmADP for isolated mitochondria [48,51]. Even more, two populations of mitochondria were found in human clinical breast cancer material: one population of mitochondria is characterized with lower KmADP (42 ± 14 μM), whereas the apparent KmADP value for the second mitochondrial population is nearly seven times higher (288 ± 67 μM) [51]. This phenomenon could be associated with the two-compartment tumor metabolism theory, which states that tumor cells function as metabolic parasites and extract energy from supporting host cells such as fibroblasts. One possible explanation for these two populations of mitochondria is derived from finding that the stromal mitochondria have higher levels of autophagy, mitophagy, glycolysis, and lipolysis, while epithelial cancer cells have means like high mitochondrial mass, OXPHOS activity, and β oxidation to utilize metabolites released by the fibroblasts [83]. This high apparent KmADP for the mitochondrial population characterized with OXPHOS (possibly fibroblasts) must be connected to VDAC channel permeability regulated by cytoskeletal elements. In contrast to that described in clinical samples, in cancer cell cultures the VDAC permeability for exogenous ADP is closer to that in isolated mitochondria. The affinity of mitochondria for ADP was high in both undifferentiated and differentiated N2a cells, HL-1 cells and also in human colorectal cancer Caco-2 cell culture (Table 1) [46,52,53,54,84,85]. The observed variation in MOM permeability between cultured cells and clinical samples indicates the profound differences between these two sample types.

## 5. Conclusions and Perspectives

Emerging evidence suggests the regulatory role of unpolymerized tubulin in cellular bioenergetics through the interaction of the β-tubulin subunit of the α/β-tubulin heterodimer with VDAC in the MOM and thereby changing channel permeability for adenine nucleotides. Numerous studies confirm the tissue specificity of the VDAC-tubulin interaction [10,20,30,37,39,52,69,86,87]. Moreover, the function of this interaction in regulation of MOM permeability is different in cell cultures, neurons, muscle, and cancer tissues. 

Clear structure-function relationships in the bioenergetics of the oxidative and glycolytic muscle cells are related to the formation of the large supramolecular complexes. In the fully formed MI complex of the oxidative muscle cells, the interaction between βII-tubulin CTT and VDAC impedes adenine nucleotide movement through the channel and works as a switch, by which energy-rich phosphate is directed to the energy transport networks (Figure 1). The changes in tissue-specific diffusion restrictions for adenine nucleotides in the level of MOM can be easily characterized by measuring the apparent KmADP in permeabilized cells and tissues [88]. Furthermore, determination of MI composition and function in cardiac cells may provide opportunities for possible bioenergetic therapy associated with reinforcement of the binding of tubulin to VDAC to maintain the regulation of mitochondrial respiration via micro-compartmentalization of adenine nucleotides and the control of energy fluxes through MOM.

The composition of MI in cancer cells is not profoundly studied. In the MI model of cancer cells unpolymerized tubulin and/or HK can interact with VDAC (Figure 1). It can also be concluded that in cancer cells the binding sites on MI components for tubulin and HK are different. It has been suggested that binding of HK to VDAC promotes aerobic glycolysis and prevents mitochondria-driven cell death [70]. Unpolymerized tubulin‒VDAC interaction controls mitochondrial membrane potential in cancer cells [71], but the mechanisms and regulation of this interaction are not clear. However, the proteins that exert control over VDAC permeability may contribute to metabolic plasticity of cancers. It is likely, however, that anti-tumor drugs modulating microtubules (e.g., taxol) might modify interactions of MTs and unpolymerized tubulin (e.g., βIII-tubulin) with mitochondria and thus significantly affect cellular energy metabolism. Therefore, influencing VDAC interactions with tubulin and HK could be a target for the new generation of cancer therapy [34,72,89].

The exact tubulin isotypes that form regulatory interactions in cancer and in other cell types are not yet known. Any description regarding the regulation of VDAC by tubulin CTTs must take into account the fact that both α- and β-tubulin CTTs are likely involved, because they both can access the VDAC pore [86,90]. Also, it is not clear if and how the globular body of the tubulin interacts with VDAC. Moreover, regulation of the MOM permeability could be related to the presence of post-translational modifications in β-tubulin, the participation of other tubulin isoforms, the interplay between energy transfer pathways, or changes in the phosphorylation state of the VDAC channel and/or tubulin. Recent findings associate gamma-tubulin and the γ-tubulin formed meshwork with mitochondrial membranes and mitochondrial function [91,92]. Whether the mitochondria‒gamma-tubulin interaction has any effect on VDAC permeability or on the recruitment of unpolymerized αβ-dimers has not been studied. Understanding the mechanisms and consequences of the tubulin interaction with VDAC is important for the identification of potential targets for VDAC and for mitochondria. 

## Figures and Tables

**Table 1:** Apparent KmADP for exogenous ADP in regulation of respiration in permeabilized cells and fibers from different tissues with or without creatine or trypsin treatment.

Tissue	Apparent KmADP ^1^, µM	References
Rat heart mitochondria	10–20	[12,21,23,26,31]
Rat heart fibers	300–500	[21,23,25,26,27,31]
Rat heart isolated cardiomyocytes	300–500	[13,14,38,46]
Trypsin-treated rat heart fibers	100–110	[21,23]
Rat heart fibers in the presence of creatine	80–100	[21,25,26,31]
Rat *m. soleus*	300-400	[21,30]
Rat *m. gastrocnemius* white	5–15	[21,30]
Rat brain mitochondria	10	[47]
Rat brain synaptosomes	110	[47]
Rat brain synaptosomes in the presence of creatine	25	[47]
Human colorectal cancer	90–130	[48,49,50]
Human breast cancer		
Mitochondrial population I	45	[51]
Mitochondrial population II	300	[51]
Cell lines		
Mouse neuroblastoma N2a	20–40	[52,53]
Mouse sarcoma HL-1	25–50	[46]
Human colorectal cancer Caco-2	40	[54]

^1^ These apparent *K*_m_ values for ADP were determined from corresponding titration curves by fitting experimental data to non-linear regression equation according to a Michaelis–Menten model.

## Abbreviations

ANT	Adenine nucleotide translocase
CTT	C-terminal tale
HK	Hexokinase
IMS	Mitochondrial intermembrane space
MI	Mitochondrial Interactosome
MT	Microtubule
MIM	Mitochondrial inner membrane
MOM	Mitochondrial outer membrane
MtCK	Mitochondrial creatine kinase
OXPHOS	Oxidative phosphorylation
VDAC	Voltage-dependent anion channel
